# Enhancing the stretchability of two-dimensional materials through kirigami: a molecular dynamics study on tungsten disulfide†

**DOI:** 10.1039/d4ra04814h

**Published:** 2024-08-05

**Authors:** K. Dey, S. Shahriar, M. A. R. Anan, P. Malakar, M. M. Rahman, M. M. Chowdhury

**Affiliations:** a Department of Mechanical Engineering, Bangladesh University of Engineering and Technology Dhaka 1000 Bangladesh koushik.dey.buet@gmail.com; b Department of Mechanical Engineering, The University of New Mexico Albuquerque NM 87131 USA; c Department of Materials Science and Engineering, University of Michigan Ann Arbor Michigan 48109 USA; d School of Electrical and Computer Engineering, Purdue University West Lafayette Indiana 47907 USA; e Department of Electrical and Computer Engineering, University of British Columbia Vancouver British Columbia V6T 1Z4 Canada; f Quantum Matter Institute, University of British Columbia Vancouver British Columbia V6T 1Z4 Canada

## Abstract

In recent years, the ‘kirigami’ technique has gained significant attention for creating meta-structures and meta-materials with exceptional characteristics, such as unprecedented stretchability. These properties, not typically inherent in the original materials or structures, present new opportunities for applications in stretchable and wearable electronics. However, despite its scientific and practical significance, the application of kirigami patterning on a monolayer of tungsten disulfide (WS_2_), an emerging two-dimensional (2D) material with exceptional mechanical, electronic, and optical properties, has remained unexplored. This study utilizes molecular dynamics (MD) simulations to investigate the mechanical properties of monolayer WS_2_ with rectangular kirigami cuts. We find that, under tensile loading, the WS_2_ based kirigami structure exhibits a notable increase in tensile strain and a decrease in tensile strength, thus demonstrating the effectiveness of the kirigami cutting technique in enhancing the stretchability of monolayer WS_2_. Additionally, increasing the overlap ratio enhances the stretchability of the structure, allowing for tailored high strength or high strain requirements. Furthermore, our observations reveal that increasing the density of cuts and reducing the length-to-width ratio of the kirigami nanosheet further improve the fracture strain, thereby enhancing the overall stretchability of the proposed kirigami patterned structure of WS_2_.

## Abstract 2

Kirigami patterns on monolayer WS_2_ enhance stretchability and tailor mechanical properties. MD simulations show increased tensile strain, reduced strength, and optimal performance from adjusted overlap ratio, cut density, and length-to-width ratios.

## Graphical abstract

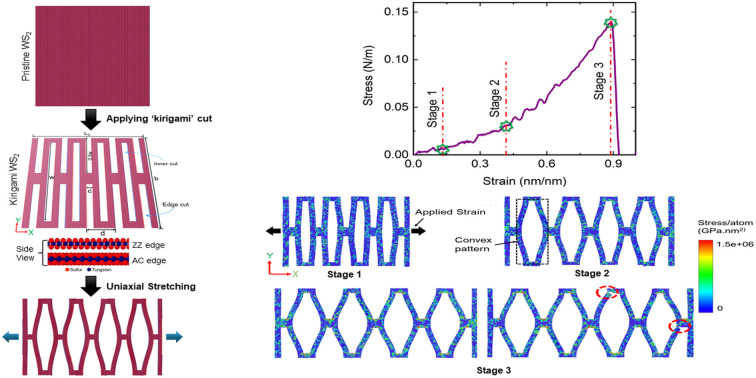

## 1 Introduction

Two-dimensional (2D) materials, including graphene, hexagonal boron nitride (h-BN), phosphorene, and transition metal dichalcogenides (TMDs), have garnered significant attention due to their favorable physical, chemical, optical, and electrical properties and diverse applications.^1–6^ Beyond their exceptional properties, the capacity to stack these materials to construct desired heterostructures without introducing interface-induced nonidealities arising from lattice mismatches not only renders 2D materials an ideal platform for exploring novel physical phenomena but also for fabricating advanced heterostructured devices.^7^ More remarkably, the incorporation of a kirigami pattern,^8^ a novel approach to nanofabrication that specifically utilizes the cutting of thin sheets, on 2D materials enables the construction of robust microscale structures with tunable mechanical properties.^9^ This kirigami patterning facilitates the creation of meta-structures and meta-materials with unique properties, such as a negative Poisson's ratio, accurate shape morphing, tunable auxetics, super-stretchability, buckling, and multistability through the modification of certain materials or structures.^10–14^ The exceptional stretchability and flexibility of kirigami metastructures have been harnessed to develop systems, equipment, macromaterials, and microstructures,^15^ revolutionizing the research and applications of 2D materials in various fields.^16^

Recent research endeavors have focused on leveraging kirigami to enhance the stretchability of 2D materials. Molecular dynamics (MD) simulations have been employed to investigate the mechanical properties of kirigami-modified 2D materials, including MoS_2_,^17,18^ graphene,^19,20^ h-BN,^21^ and phosphorene^22^ under various conditions. In particular, the application of kirigami engineering was first explored with graphene which has led to a significant improvement in fracture strain properties at nanoscale, nearly three times greater than those observed in pristine monolayer graphene.^19,23^ Similarly, the kirigami techniques have led to substantial enhancements in the mechanical properties of MoS_2_ (fourfold increase in yield strain and sixfold increase in fracture strain), h-BN (three to five times increase in fracture strain), and phosphorene (almost twofold increase in fracture strain) compared to their pristine counterparts.^24–26^

Experimentally, kirigami can be achieved in a number of ways including photolithography, chemical vapor deposition, oxygen plasma etching, two-photon polymerization (2PP) *etc.*^9,27,28^ The first experimental demonstration of kirigami was conducted by Blees *et al.* where pristine graphene sheets were patterned using conventional photolithography techniques.^9^

Following that, the technique of kirigami was also applied to TMDs using CVD and etching-based methods.^27,29^ In both cases, kirigami resulted in enhanced stretchability and transport properties with potential applications in the field of stretchable electronics. Similar top-down approach have been employed to fabricate kirigami MoS2, 2D PtSe2, *etc.* materials as well.^30^ Tungsten disulfide (WS_2_), a two-dimensional material, has gained prominence in various applications such as gas sensing applications, optical modulators, solid and dry film lubricants and as self-lubricating composite materials.^31^ It exhibits promising potential in the fields of nanoelectronics, spintronics, and optoelectronics, owing to its desirable properties such as a 2 eV energy gap between bound and free states, low heat conduction, and strong spin–orbit interaction.^32^ Notably, WS_2_ demonstrates better tribological properties and heat resistance than MoS_2_, making it a viable alternative for similar applications.^31^ By extending the kirigami approach to enhance the stretchability of monolayer pristine WS_2_, exciting possibilities may emerge for its utilization in stretchable and wearable electronics.^33^

The aim of this study is to investigate the efficacy of the kirigami concept in improving the stretchability of WS_2_, a 2D material with exceptional electrical,^34^ mechanical,^35^ chemical,^36^ and optical^37^ properties. In this study, rectangular kirigami cuts are introduced to pristine monolayer WS_2_ nanosheets. Then, molecular dynamics (MD) simulations are employed to analyze the impacts of kirigami cuts on the stretchability of the structure under uniaxial tensile loading. A comprehensive comparison of stress–strain responses between kirigami WS_2_ and its monolayer pristine counterpart is presented. Furthermore, the influence of chiral orientations (armchair (AC) and zigzag (ZZ)), overlap ratio, cut density and nanosheet length-to-width ratio on the mechanical parameters of the kirigami structure, including ultimate tensile strength (UTS), and fracture strain, are investigated. Since, the fabrication techniques of kirigami cut materials mostly involve fabrication methods^28^ related to complex chemistry such as chemical vapor deposition,^27^ photolithography, oxygen plasma etching, two-photon polymerization (2PP) *etc.*, the outstanding computational results will certainly motivate the researchers for the fabrication of the kirigami structures. The results will be valuable not only to the field of computational nanomaterial chemists but to the 2D material experimental community.

## 2 Models and simulation methods

**Fig. 1:**
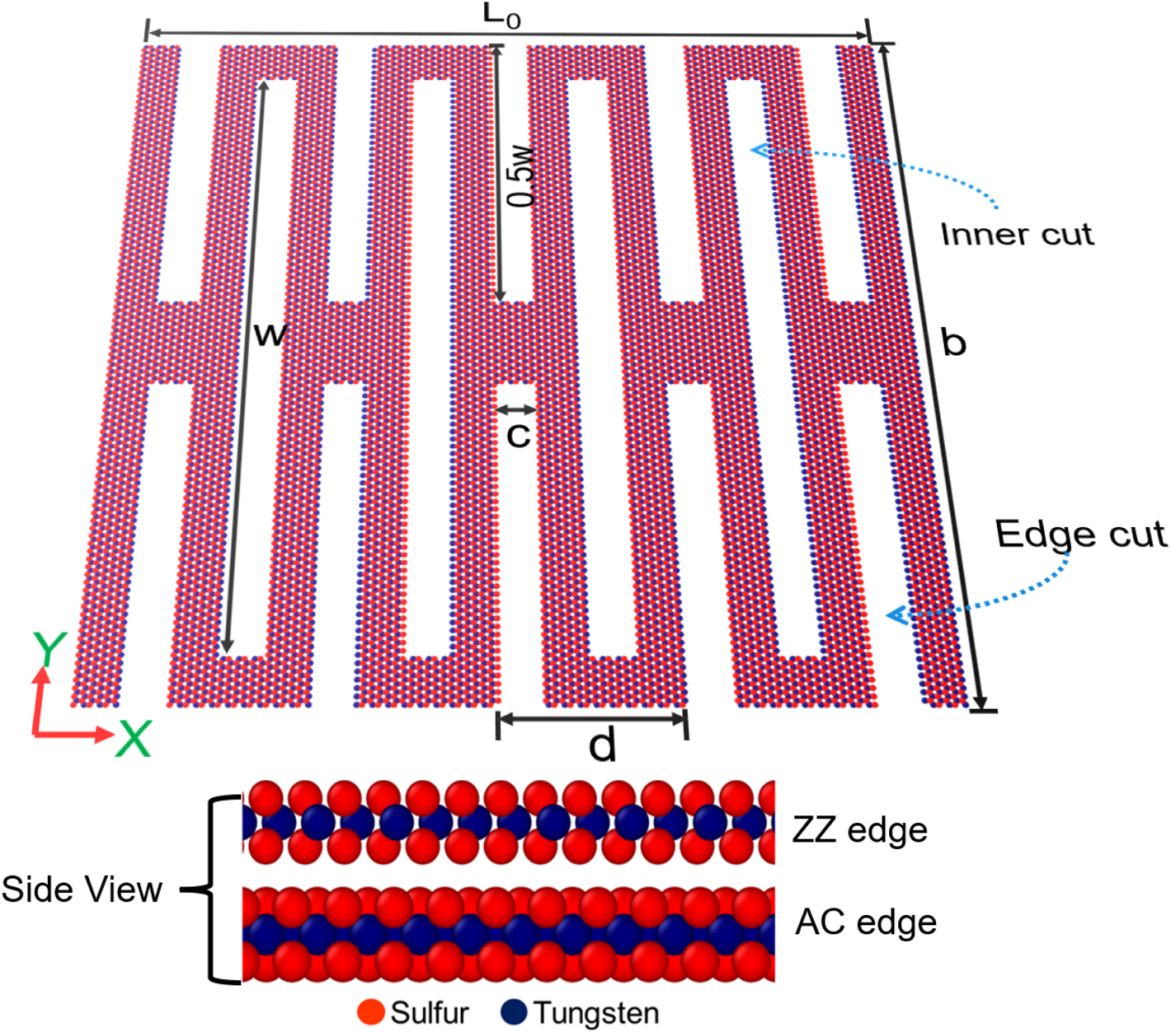
Snapshot of the top view of a monolayer kirigami WS_2_ nanosheet (*α* = 0.38, *β* = 0.045 and *γ* = 1) labeled with essential geometric parameters. The armchair (AC) and zigzag (ZZ) edges are shown from the side views of the nanosheet.

The WS_2_ kirigami sheets were created by applying a different number of rectangular kirigami cuts (inner cuts and edge cuts).to a monolayer pristine WS_2_ nanosheet. A schematic top view of the kirigami WS_2_ structure consisting of four inner cuts and five pairs of edge cuts, and the related geometric parameters is shown in Fig. 1 along with the AC and ZZ edges (side views) in the model. The main geometric parameters are the nanosheet length *L*_0_, width *b*, height of each inner cut *w*, height of edge cuts 0.5*w*, the width of each inner cut *c*, and the distance between successive cuts *d*. Following the framework developed in earlier works,^17,19,21,22^ the overlap ratio, *α*, is defined as the overlapping cut length to the nanosheet length, given by *α* = (*w* − 0.5*b*)/*L*_0_. Similarly, the ratio of the overlapping width to the nanosheet length is defined as *β* = (0.5*d* − *c*)/*L*_0_. Additionally, the length-to-width ratio of the nanosheet is denoted as *γ* = *L*_0_/*b*. *α* and *β* parameterize the fraction of rectangular voids (%v) in the kirigami structures. Unless mentioned otherwise, we varied *w* and *c* while keeping the other parameters (*L*_0_, *b*, *d*) constant in order to vary *α* and *β*, respectively. As reported in literature,^17,19^ while *α* describes %v perpendicular to the tensile loading direction, *β* describes %v parallel to the tensile loading direction. It is noticeable that *w* → 0 corresponds to pristine WS_2_ with *α* = −*b*/2*L*_0_ whereas *w* → b leads to kirigami WS_2_ with *α* = *b*/2*L*_0_. Again, *c* → 0 corresponds to pristine WS_2_ with *β* = *d*/2*L*_0_, whereas *c* → 0.5*d*, leads to kirigami WS_2_ with *β* = 0. Therefore, for realistic kirigami structure, we can consider these limits −*b*/2*L*_0_ < *α* < *b*/2*L*_0_ and 0 < *β* < *d*/2*L*_0_.

The value of *γ* is varied by changing *L*_0_ while keeping the number of kirigami cuts constant. ESI materials† contain a GIF that illustrates the evolution of the kirigami structure in relation to *α*, *β*, *γ*. In this study, we considered kirigami for both AC and ZZ edges. Fig. 1 illustrates a representative AC WS_2_ kirigami, comprising *N* = 18 984 atoms, with a nanosheet length *L*_0_ ≈ 300 Å, width *b* ≈ 300 Å, height of each inner cut *w* ≈ 265 Å, width of each inner cut *c* ≈ 19 Å, and distance between successive cuts *d* ≈ 65 Å.

The MD simulations on the kirigami were conducted employing the widely used open-source code LAMMPS,^38^ developed by Sandia, while incorporating the Stillinger–Weber (SW) potential specifically tailored for WS_2_ as proposed by Jiang *et al.*^39^ The calibration of this potential was based on the phonon dispersion phenomena of single-layer 1H-WS_2_, which encompasses both out-of-plane and in-plane vibrational motions.^40^ So, the SW potential is expected to effectively capture out-of-plane deflections involving changes in angles and rotations.^39^

The interatomic potential model employed in the present work is very useful in describing the dynamics of a wide range of systems including the 2D kirigami systems.^41–44^

The structural and energetic evolution of single-layer 1H-WS_2_ predicted by the SW potential is proven to be accurate in the literature.^17,35,39,41^ The theoretical studies of single-layer 1H-WS2 have largely utilized first-principles calculations. The SW potential, derived from a VFF model, which includes bond stretching and angle bending interactions fitted to phonon dispersion data matches the results. The process of applying tensile strain by keeping the ends fixed is also widely accepted and the deformation geometry predicted by this potential matches well with the other research works available in literature.^17,35,39,41^ In addition, the Young's modulus and Poisson's ratio was found to be in line with *ab initio* results.^45,46^ The nonlinear elastic constant and ultimate stress values also showed good agreement with theoretical predictions. And therefore, it is very suited for simulating mechanical behaviors of kirigami cut WS_2_.

In the simulation procedure, after energy minimization, NVE, NPT and NVT ensembles were applied to the system for 50 ps, 100 ps and 20 ps respectively for equilibration at a timestep of 1 fs. Then, uniaxial tensile strain was applied to the two opposing edges of the nanosheet with a 10^9^ s^−1^ strain rate maintaining periodic boundary conditions in every direction. The stresses were estimated using virial stress theorem.^47^ The structures were created using Atomsk^48^ and atomic visualizations were done with the OVITO package.^49^ In the context of 2D materials, where thickness is atomically thin and constant, stress is referred to as normalized by the unit thickness, measured in units of N m^−1^.^17,19^

## 3 Results and discussion

**Fig. 2:**
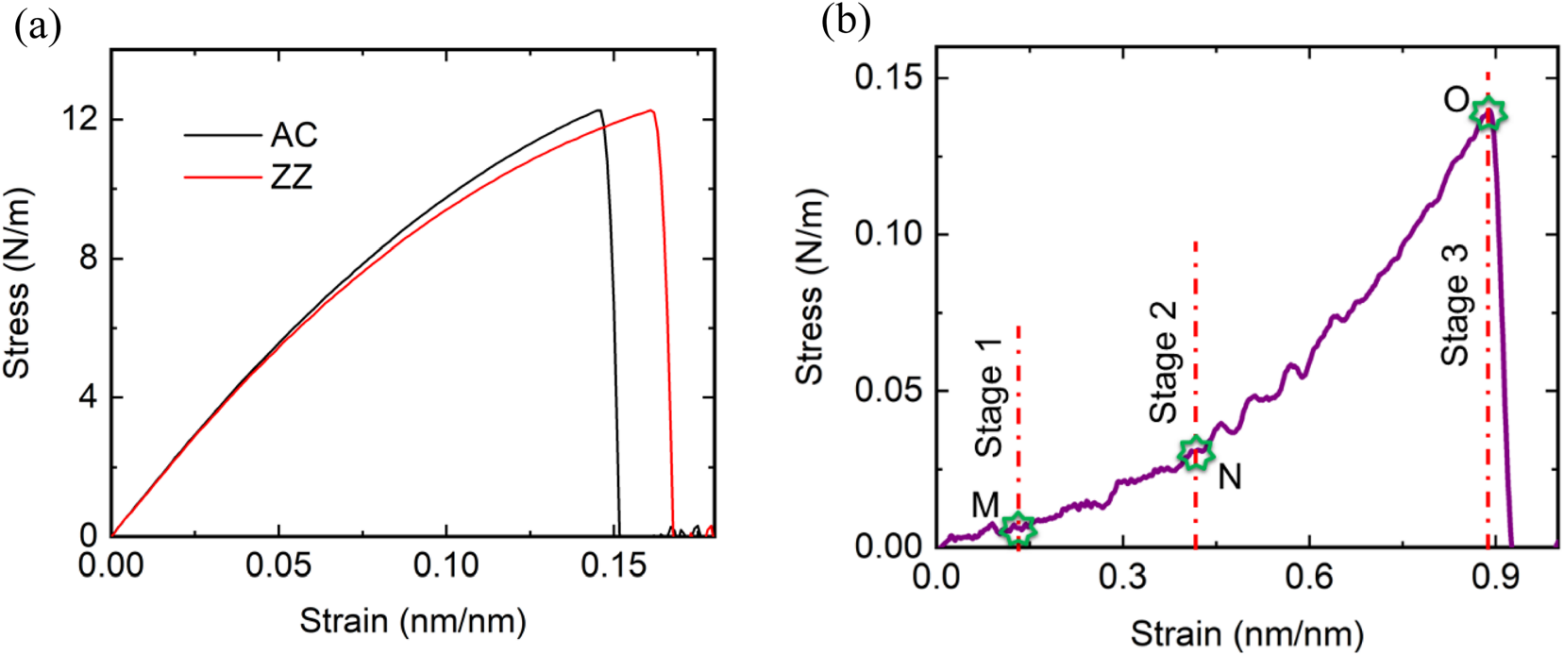
(a) Stress–strain responses of monolayer pristine WS_2_ nanosheet along AC and ZZ edges. Fracture strain obtained along ZZ edge is higher than that of obtained along AC edge. (b) Stress–strain curve of kirigami engineered monolayer WS_2_ nanosheet (*α* = 0.38, *β* = 0.045 and *γ* = 1) with 3 deformation stages when applied uniaxial tensile strain along AC edge.

We start by calculating the stress–strain response of a monolayer pristine WS_2_ nanosheet (300 Å × 300 Å) to both benchmark our simulator as well as to obtain a comparison for the kirigami-cut nanosheets. Fig. 2(a) shows how the stress in pristine WS_2_ varies with applied uniaxial tensile strain along the AC and ZZ edges. Similar to the previous studies,^50,51^ we have observed anisotropic mechanical characteristics when applying uniaxial tensile strain along the AC and ZZ edges. Our study shows that pristine WS_2_ can undergo tensile strain of up to approximately 17%, with slightly higher stretching observed along the ZZ edge compared to the AC edge.

At the point of peak stress, we have found the UTS to be 12.61 N m^−1^ along the AC edge, slightly higher than that observed along the ZZ edge. To determine Young's modulus, we employed Hooke's law and linearly fitted the stress–strain curve at low strain levels (<0.2%). The calculated Young's modulus values are 90.16 N m^−1^ for the AC edge and 88 N m^−1^ for the ZZ edge. For the specific nanosheet size of 54.2 Å × 62.6 Å, which has also been used in a previous study,^41^ our findings are consistent with the literature, as summarized in Table 1. The agreement between our findings and the existing literature underscores the accuracy of our simulator.

**Table 1:** Mechanical characteristics of monolayer pristine WS_2_ under uniaxial tensile loading

Material direction	Fracture strain	UTS (N m^−1^)	Young's modulus (N m^−1^)
AC (current study)	18.1%	13.77	114
AC^41^	18.63%	14	115
ZZ (current study)	22%	13.73	111.8
ZZ^41^	23%	13.9	113.2

In comparison to the pristine structure (Fig. 2(a)), the kirigami structure demonstrates enhanced stretchability under applied tension at the AC edge of the model, as illustrated in Fig. 2(b). In this case, the kirigami structure had an overlap ratio of *α* = 0.38, and the simulation was conducted at a temperature of 300 K. To closely examine the impact of the kirigami cutting method on the mechanical properties of monolayer WS_2_, we analyze the three deformation stages of the corresponding structure, as shown in Fig. 2(b). The ending of deformation stages 1, 2 and 3 are marked with points M, N and O, respectively.

In stage 1, a slight increment in stress (∼0.005 N m^−1^) is observed at the onset of deformation as the strain reaches up to 15%. Progressing into stage 2, the stress rises to ∼0.029 N m^−1^ with further strain increase from 15% to 43%. Transitioning to stage 3, a substantial surge in stress (∼0.14 N m^−1^) occurs as the strain continues to rise from 43% to 88.3%. The deformation stages observed in our study are consistent with those reported in a previous study of hBN kirigami by Gamil *et al.*^22^

**Fig. 3:**
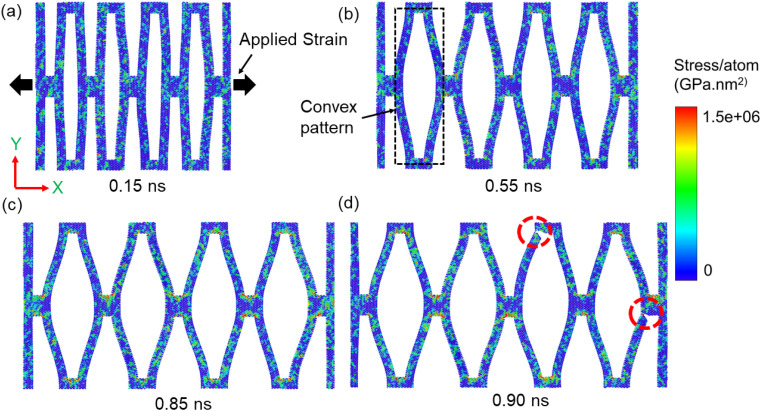
Atomic visualization of the deformation process of a kirigami WS_2_ nanosheet (*α* = 0.38, *β* = 0.045 and *γ* = 1) at various timestamps: (a) 0.15 ns, (b) 0.55 ns, (c) 0.85 ns and (d) 0.90 ns, at a temperature of 300 K. The kirigami nanosheet is subjected to uniaxial tensile strain along the AC edge (*x*-direction). At 0.90 ns, cracks (indicated in red circles) are clearly visible both at internal and external portion of rhomboid structure. The color coding in the figure represents per atom stress, ranging from 0 to 1.5 × 10^6^ GPa nm^2^.

In order to further understand the effect of kirigami cut, we delve into a more detailed examination of the three deformation stages observed here. Fig. 3 illustrates the atomic visualization of the deformation process of the kirigami sheet (*α* = 0.38, *β* = 0.045 and *γ* = 1) at various timestamps of 0.15 ns, 0.55 ns, 0.85 ns and 0.90 ns. The kirigami sheet is subjected to uniaxial tensile strain along the AC edge (*x*-direction). The color coding in the figure represents per atom stress, ranging from 0 to 1.5 × 10^6^ GPa nm^2^.

In deformation stage 1, the kirigami structure expands by approximately 7%, with the inner cuts (denoted by *w* in Fig. 1) exhibiting slight elongation along the loading direction (Fig. 3(a)). The stress increment in stage 1 is negligible (∼0.005 N m^−1^). In this stage, the deformation mechanism primarily involves elastic bond stretching, while no flipping or rotation of the WS_2_ kirigami sheet is observed. This is in contrast to previous studies on graphene kirigami,^19^ where rotation and flipping were observed in the initial stage. Hanakata *et al.*^17^ reported that the absence of such behavior could be attributed to the higher elastic bending modulus of the structure under investigation compared to monolayer graphene.^52^

Transitioning to stage 2, we have observed substantial out-of-plane deformation of the rectangular cuts under the applied tension. Simultaneously, the cuts undergo a transformation, gradually deforming into convex like patterns (*i.e.*, middle section is thicker than edges).^13,53^ Within this stage, the stress exhibits a notable increase from 0.005 N m^−1^ to 0.029 N m^−1^ as the strain progressively rises from 7% to 43%. The emergence of the convex pattern becomes clearly visible after 0.55 ns, as shown in Fig. 3(b).

In stage 3, as the structure undergoes further stretching along the loading direction, the out-of-plane deformation of the inner cuts gradually diminishes. As the applied load causes the kirigami sheet to stretch further, it becomes nearly flat. Notably, the convex pattern persists even at 0.85 ns of deformation (Fig. 3(c)). The stress within the structure reaches a significant level (∼0.14 N m^−1^), resulting in stress concentration at the mechanical joints of the cuts, as indicated by the color code in Fig. 3. Eventually, fracture occurs at a strain of 88.3% as shown in Fig. 3(d). The crack point, denoted by O in Fig. 2(b), corresponds to the location of maximum stress (∼0.14 N m^−1^) which is also indicated by the dashed red circles in Fig. 3. Within a narrow strain range of approximately 2%, the kirigami structure experiences complete rupture following the initiation of the crack.

**Fig. 4:**
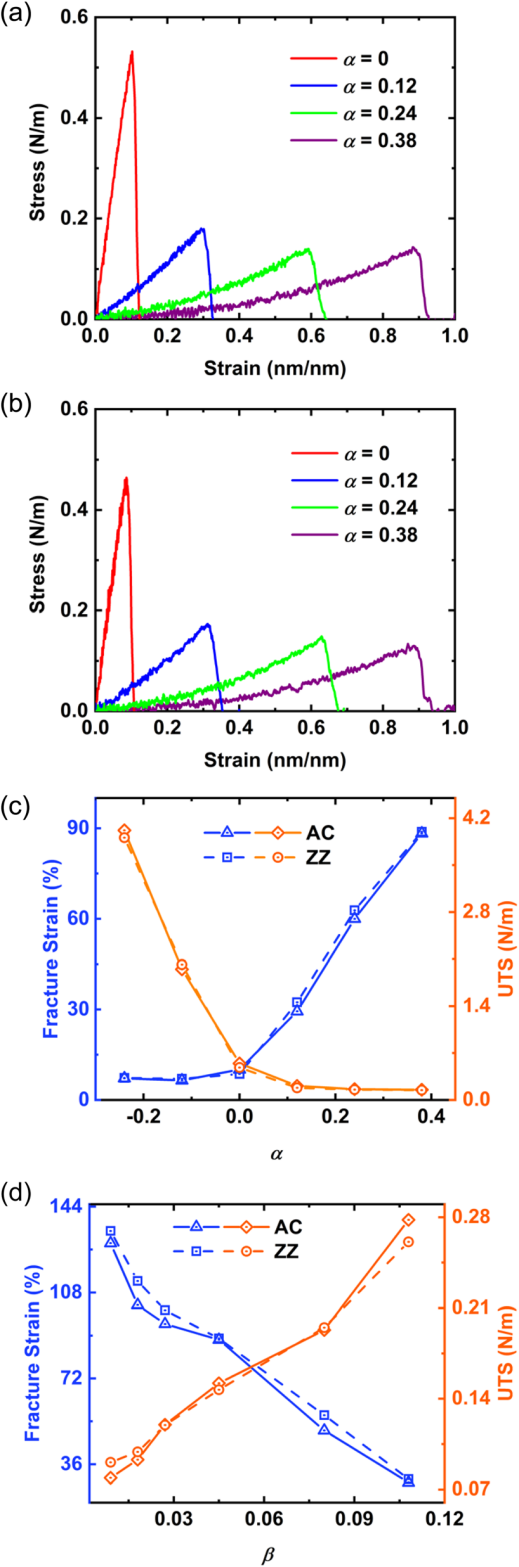
Stress–strain curves of kirigami WS_2_ with different overlap ratios (*α*) and constant *β* = 0.045, *γ* = 1 under uniaxial tensile strain at 300 K temperature along the (a) armchair (AC) edge and (b) zigzag (ZZ) edge. Variation of fracture strain and UTS with (c) overlap ratio, *α* and (d) *β*.

Next, we analyze the effect of *α*, *β* and *γ* on the mechanical characteristics of the kirigami structures. Fig. 4 shows the influence of the overlap ratio (*α*) on the stress–strain behavior of kirigami-WS_2_ models along the AC and ZZ edges. Increasing the cut length, denoted as *w* in Fig. 1, leads to higher values of *α*, and *vice versa*. Fig. 4 indicates that by controlling the overlap ratio, we can effectively manipulate the stretchability and tensile strength of the kirigami nanosheet. Under the tensile loading along the AC edge, as *α* increases from 0 to 0.38, the fracture strain increases from ∼10.1% to ∼88.3%, while the UTS decreases from ∼0.545 N m^−1^ to ∼0.152 N m^−1^. Similarly, along the ZZ edge, increasing *α* from 0 to 0.38 leads to an increase in fracture strain from ∼8.6% to ∼88.8%, accompanied by a decrease in UTS from approximately ∼0.484 N m^−1^ to ∼0.147 N m^−1^. The ZZ edge exhibits a relatively lower UTS, but a slightly higher fracture strain compared to that of along the AC edge. The variation in stress response along different chiral orientations can be attributed to the bond structures^54^ of WS_2_.

WS_2_ features a trigonal-prismatic crystal structure known as h-WS_2_, where a layer of tungsten atoms is sandwiched between two layers of sulfur atoms. In the ZZ edge, the S–W–S and S–W–S′ bonds are densely packed compared to the AC edge, where S and S′ belong to different groups on the top or bottom side.^55^ The bond angles along the ZZ edge can stretch further before fracture under tensile loading.^41^ This distinction may provide a possible explanation for the slightly higher fracture strain observed when loaded along the ZZ edge as compared to the AC edge.

The deformation mechanism observed at different *α* values can be explained as follows. *α* = 0 represents the configuration where the edge and inner cuts begin to overlap (Fig. 1). Han *et al.*^21^ reported that when *α* is less than 0, the edge and inner cuts do not overlap, severely constraining or even inhibiting out-of-plane deformation at lower *α* values. In this scenario, the cuts can be regarded as atomic line defects, which can significantly reduce the strength and strain of the structure. Smaller values of *α* correspond to higher UTS, approaching that of the cut-free nanosheet. Similar kind of observations were made for *α* values of −0.24, −0.12 and 0. Hence, our analysis indicates that at lower overlap ratios (*α* ≤ 0), the desired advantages of the kirigami patterned cut, such as high stretchability, cannot be achieved. This phenomenon has been observed in previous studies on various materials, such as kirigami graphene,^19^ MoS_2_,^17^ hBN^21^ and phosphorene.^22^

In contrast, higher overlap ratios may lead to enhanced stretchability. Our study indicates that increasing the *α* value from 0.12 to 0.38 resulted in a decrease in stress from 0.21 N m^−1^ to 0.152 N m^−1^, while the fracture strain increased from 29.4% to 88.3%. Han *et al.*^21^ reported that when *α* is greater than 0, the flipping and rotation mechanism at stage 2 and 3 becomes prominent. This mechanism significantly contributes to the extensive stretchability of the kirigami configuration. With an increasing overlap between the edge and interior cuts, the stretchability of the kirigami WS_2_ structure progressively increases.^17,19,21,22^

Next, we investigated the influence of *β* on the stretchability of the kirigami nanosheet. As highlighted before, the value of *β* is changed by altering *c* while all other geometric factors remain unchanged. The results presented in Fig. 4(d) demonstrate that, under the tensile loading along the AC edge, an increase in *β* from 0.009 to 0.108 corresponds to a decrease in fracture strain from approximately 128.7% to 28.4%, while the UTS shows an increase from about 0.079 N m^−1^ to 0.278 N m^−1^. Likewise, along the ZZ edge, an increase in *β* from 0.009 to 0.108 is accompanied by a decrease in fracture strain from approximately 133.8% to 29.8%, along with an increase in UTS from around 0.091 N m^−1^ to 0.261 N m^−1^. This behavior can be attributed to the fact that, when *β* values are higher, the density of cuts decreases, resulting in kirigami structures that exhibit characteristics almost similar to pristine structures. Consequently, this leads to lower fracture strain and higher UTS. Because of the same reason, we observe almost similar scenarios along ZZ edges when *β* is higher (Fig. 4(d)).

The observed effects of *α* and *β* in this study aligns with previous investigations on kirigami graphene,^19^ MoS_2_,^17^ hBN^21^ and phosphorene.^22^ As we mentioned earlier, in a study exploring the effects of various parameters on the mechanical properties of kirigami MoS_2_,^17^ the authors reported an increase in fracture strain by a factor of 6 compared to its pristine counterpart. In our study, for the kirigami structure with *α* = 0.38, *β* = 0.009 and *γ* = 1, we have observed an increase in fracture strain by a factor of ∼9 along the AC direction, providing evidence that kirigami significantly enhances stretchability of monolayer WS_2_. As highlighted before, the kirigami structure enables a significant trade-off between stress and strain. Higher stretchability is accompanied by a lower fracture stress point, and *vice versa*. This trade-off is evident in the trends observed in Fig. 4(c) and (d), illustrating that an increase (decrease) in *α* (*β*) results in an increase in fracture strain and a decrease in UTS. In other words, increase (decrease) in *α* (*β*) enhances the impact of kirigami.

**Fig. 5:**
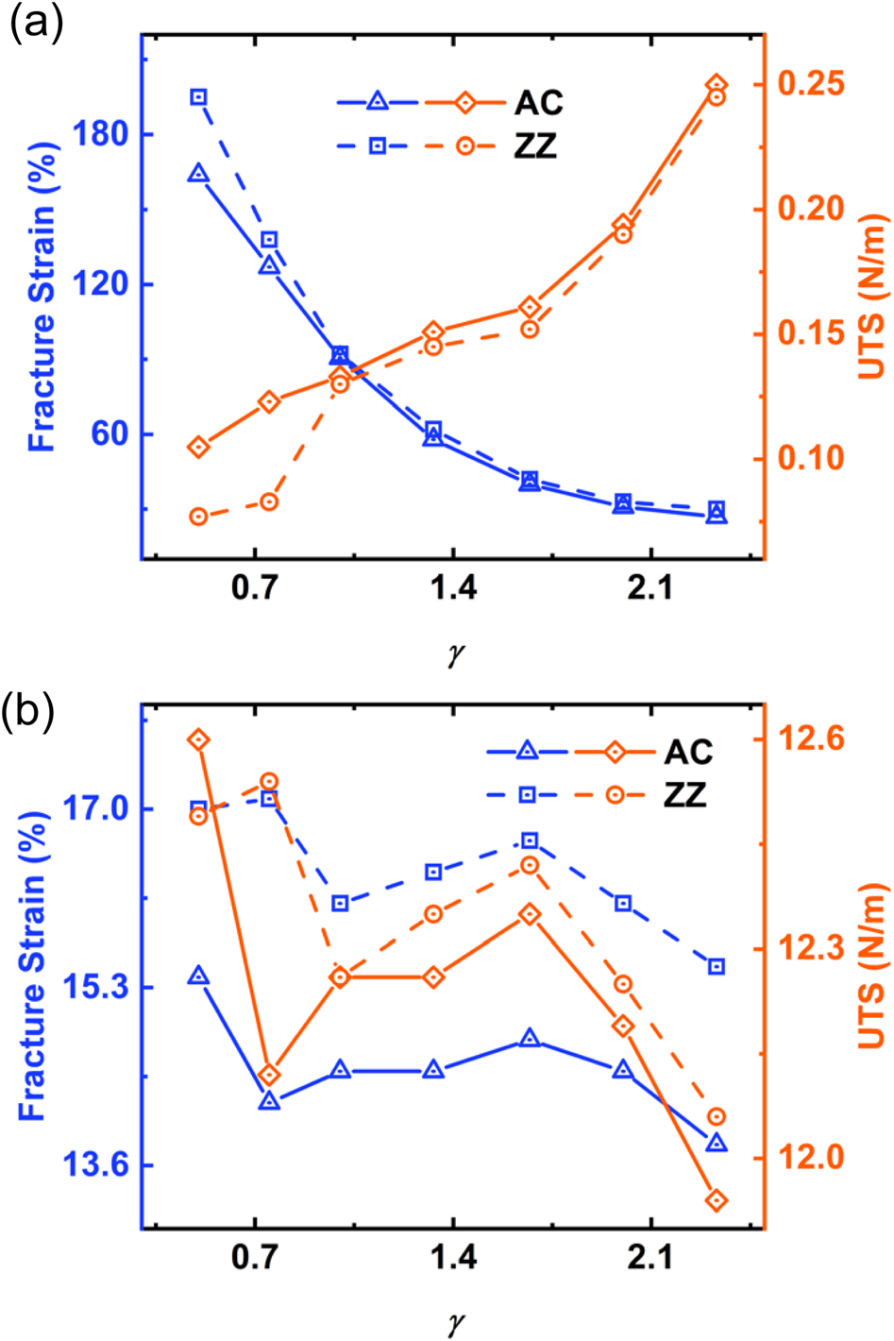
Influence of length-to-width ratios (*γ*) on the fracture strain and UTS of (a) kirigami WS_2_ (*α* = 0.38, *β* = 0.045) (b) pristine WS_2_ nanosheets under uniaxial tensile strain along AC and ZZ directions.

Now, to examine the impact of the length-to-width ratio (*γ*), we varied *γ* while keeping *α* constant. Our findings reveal that, under the tensile loading along the AC edge, as *γ* decreases from 2.33 to 0.5, the fracture strain (Fig. 5(a)) increases from ∼27% to ∼164%, while the UTS decreases from ∼0.25 N m^−1^ to ∼0.105 N m^−1^. Similarly, along the ZZ edge, decreasing *γ* from 2.33 to 0.5 leads to an increase in fracture strain from ∼30% to ∼195%, accompanied by a decrease in UTS from ∼0.245 N m^−1^ to ∼0.077 N m^−1^. In our investigation, we increased the value of *γ* while keeping the number of kirigami cuts constant, resulting in a higher value of *d* and a lower density of cuts. This reduction in cut density caused the kirigami structure to display characteristics more akin to pristine structures, consistent with previous results shown in Fig. 4(d). As a result, the fracture strain decreased and the UTS increased with respect to *γ* (Fig. 5(a)). Additionally, the differences in behavior between the AC and ZZ edges became less pronounced as *γ* increased (Fig. 5(b)), owing to the same underlying mechanism. Our findings indicate that, in addition to *α* and *β*, we can also maximize the fracture strain by reducing the length-to-width ratio (decreasing *γ*).

We simulated monolayer pristine WS_2_ with varying length to width ratio to investigate the impact of *γ* on fracture strain and UTS (Fig. 5(b)). In contrast to the kirigami structure, we did not observe significant changes in fracture strain or UTS in the case of monolayer pristine WS_2_ when varying *γ* values. Decreasing *γ* from 2.33 to 0.5 led to an 11.6% increase in fracture strain and a 5.5% increase in UTS along the AC edge. Similarly, along the ZZ edge, we observed a 9.7% increase in fracture strain and a 3.56% increase in UTS. On the other hand, for the kirigami structures (Fig. 5(a)), decreasing *γ* from 2.33 to 0.5 resulted in a more than 500% increase in fracture strain and a 69% decrease in UTS. Fig. 5(a) illustrates that the fracture strain differences along the AC and ZZ edges are approximately 30% when *γ* is around 0.5, and both the strain values are approximately 11 times higher than those of their pristine structures as shown in Fig. 5(b). We also investigated the size effect for the pristine and kirigami nanosheets. We found that for the same *γ*, with pristine WS_2_ nanosheets of different sizes, the fracture strain and UTS do not vary that much, whereas kirigami counterparts vary substantially with the change of nanosheet sizes. The curve displaying variation of fracture strain and UTS with nanosheet size is shown in ESI Fig. 1 in the ESI Section.†

According to our discussion it is now evident that the interaction between *α*, *β*, and *γ* in kirigami-modified nanosheets involves a complex interplay that significantly impacts the material's mechanical properties. *α*, which represents the overlap ratio between middle cuts and edge cuts, enhances ductility by facilitating the flipping and rotating of cuts during deformation. However, the effectiveness of *α* is moderated by *β*, which defines the density and spacing of the cuts. Higher *β* values, indicating more frequent cuts, can distribute stress more evenly but also introduce more stress concentration points, potentially leading to earlier failure. Therefore, while a higher *α* promotes ductility, an optimal *β* is necessary to avoid excessive stress concentrations. *γ*, the length-to-width ratio of the nanosheet, further influences this interaction by affecting the overall stress distribution and deformation behavior. A higher *γ*, implying a longer and narrower sheet, enhances the effectiveness of *α* by allowing more uniform stress distribution along the sheet's length, making the flipping and rotating mechanisms more effective. Conversely, a lower *γ* can concentrate stress in smaller areas, negating some of the benefits provided by an optimal *α* and *β* combination. Thus, the interaction between these parameters must be carefully balanced: higher *α* values enhance ductility, but this must be supported by an optimal *β* to prevent stress concentrations, and an appropriate *γ* to ensure uniform stress distribution and effective deformation mechanisms. This intricate interplay is essential for achieving desired mechanical properties in kirigami-modified nanosheets, making them suitable for advanced applications that require high flexibility and strength.

The stress–strain curves are also sensitive to temperature as seen in the stress–strain graphs (ESI Fig. 2†) at different temperatures from 100 K to 600 K. At lower temperatures, the structure exhibits higher strength and can endure more stress before failing. As the temperature increases the atomic vibrations, thermal expansion, and dislocation mobility of the material also increase, leading to decreased strength and earlier failure. These changes might result from the weakening of atomic bonds and potential microstructural alterations due to the increased thermal energy. Further discussions have been added to the ESI.†

## 4 Conclusions

This study employs molecular dynamics simulations to investigate the mechanical properties of monolayer WS_2_ with rectangular kirigami cuts. Under tensile loading, our findings have revealed a notable increase in tensile strain and a decrease in tensile strength along both the AC and ZZ edges in the kirigami structure, thus demonstrating the effectiveness of the kirigami cutting technique in enhancing the stretchability of monolayer WS_2_. Similar to other kirigami-cut 2D materials, we have observed that kirigami patterning enables significant out-of-plane deformations in monolayer WS_2_, thereby facilitating tensile stretching of the structure. Furthermore, we have observed that increasing the overlap ratio (*i.e.*, increase in *α*) and augmenting the density of cuts (corresponding to a decrease in *β*) contribute to enhanced stretchability in the structure. Our study reveals that, for the WS_2_-based kirigami structure with *α* = 0.38, *β* = 0.009 and *γ* = 1, the fracture strain can undergo a nearly ninefold increase, while the corresponding UTS exhibits a decrease by a factor of 155 compared to the pristine counterpart. Additionally, for the kirigami structure, decreasing the length-to-width ratio (*γ*) 4.6 times, while keeping the number of kirigami cuts constant, resulted in a more than 500% increase in fracture strain and a 69% decrease in UTS. These tradeoffs and the broad range of values provide suitable engineering opportunities, allowing for the selection of appropriate *α*, *β* and *γ* values based on the desired mechanical stretchability and strength requirements. Overall, the results from our study demonstrate the effectiveness of the kirigami cutting technique in enhancing the stretchability of monolayer WS_2_, thus highlighting the potential of this technique in the fields of stretchable and wearable electronics. The study will also motivate novel chemical synthesis mechanisms to fabricate kirigami cut WS_2_ materials.

## Data availability

The authors confirm that the data supporting the findings of this study are available within the article [and/or] its ESI materials.†

## Conflicts of interest

There are no conflicts of interest to declare.

## Supplementary Material

RA-014-D4RA04814H-s001

RA-014-D4RA04814H-s002
