# Supplementary figures and images for: Enhancing the stretchability of two-dimensional materials through kirigami: a molecular dynamics study on tungsten disulfide
Source: RSC Adv. 2024 Aug 5;14(34):24483–91. doi: 10.1039/d4ra04814h (PMC11299158; doi:10.1039/d4ra04814h)

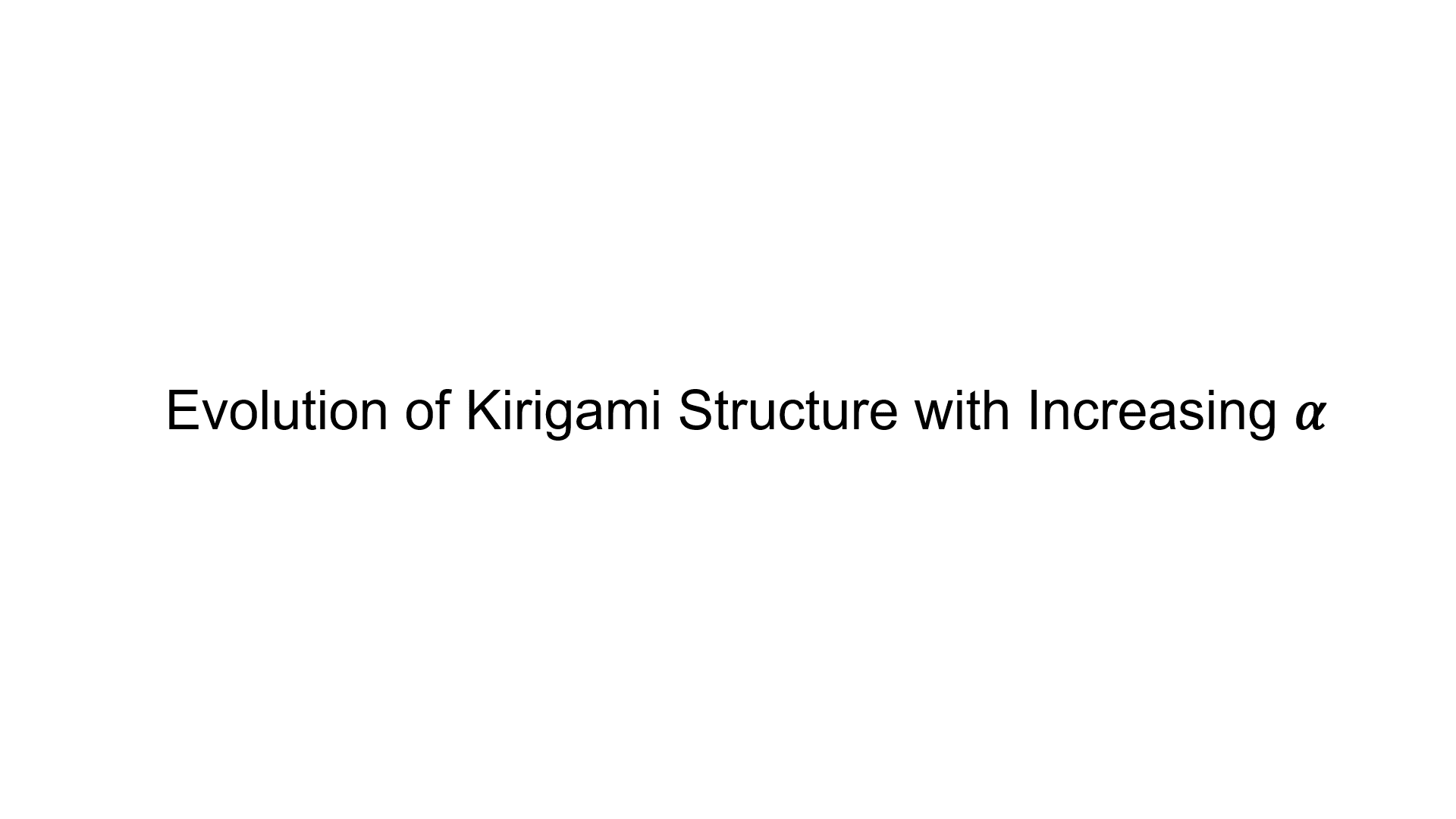

Supplement: RA-014-D4RA04814H-s001 [file RA-014-D4RA04814H-s001.gif]
