# Supplementary material for: Enhancing the stretchability of two-dimensional materials through kirigami: a molecular dynamics study on tungsten disulfide
Source: RSC Adv. 2024 Aug 5;14(34):24483–91. doi: 10.1039/d4ra04814h (PMC11299158; doi:10.1039/d4ra04814h)
Supplement: RA-014-D4RA04814H-s002 [file RA-014-D4RA04814H-s002.pdf]

Supplementary Information (SI) for RSC Advances.
This journal is © The Royal Society of Chemistry 2024

Supplementary

Size Effect:

For $\gamma = 2$, variation of Fracture Strain and UTS with Nanosheet Size in the pristine and kirigami structures is shown in the graph below:

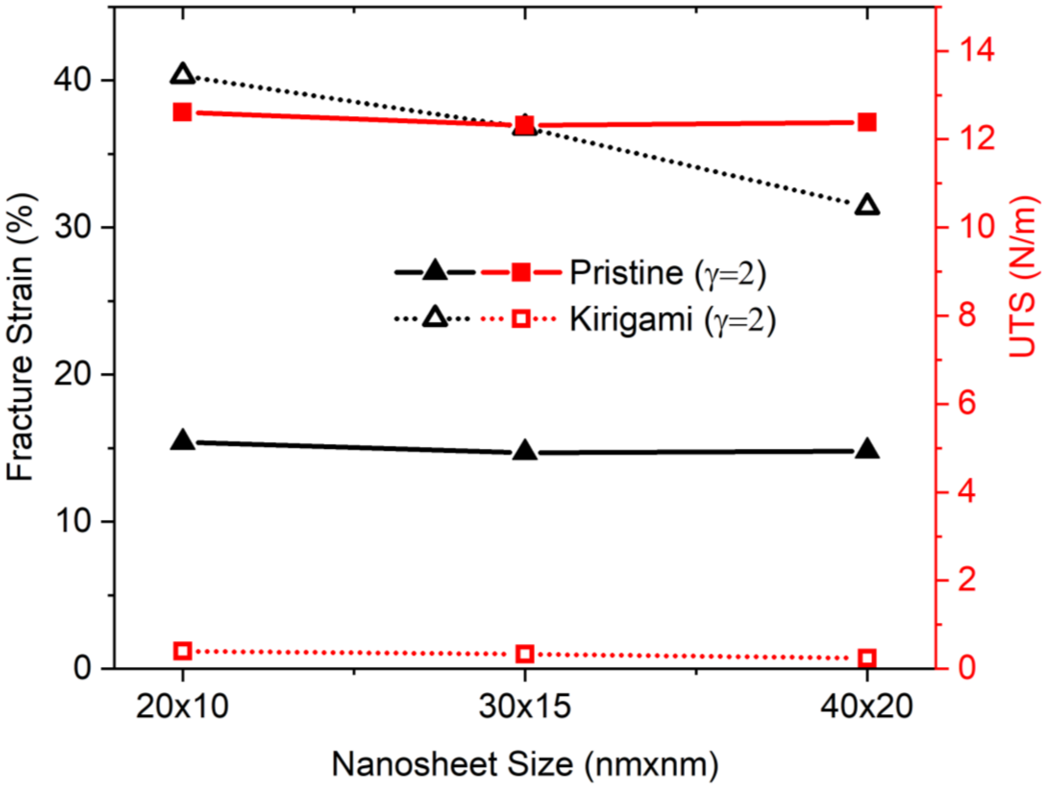

Figure 1: Variation of Fracture Strain and UTS of pristine and kirigami WS_2 having same γ=2 with nanosheet size (length (nm) X width (nm))

We can observe that for pristine WS_2 nanosheets of different sizes, the Fracture Strain and UTS do not vary that much for the same value of γ =2, whereas kirigami counterparts vary substantially with the change of nanosheet size.

Effect of temperature on the stress-strain curves:

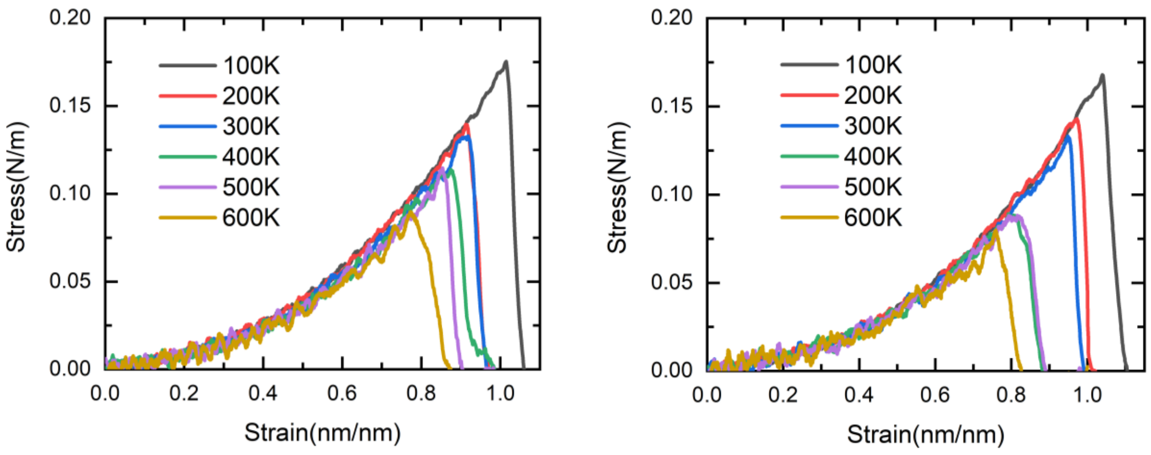

(a) **(b)**

Figure 2: Variation of Stress with Strain for kirigami WS_2 nanosheet with $\alpha = 0.38$, $\beta = 0.045$ and $\gamma = 1$ along (a) Armchair and (b) Zigzag orientations under uniaxial tensile strain at different temperatures from 100K – 600K

From Figure 2, we observe that the stress-strain curves for both chiralities are sensitive to the temperature variations. With the increase of temperature from 100K to 600K, the Ultimate Tensile Strength and Fracture Strain decrease. At the specified temperature range, the fracture strain falls from 105% to 87% in the AC orientation and the fracture stress decreases from 0.17 N/m to 0.08 N/m. In the case of ZZ orientation, the strain decreases from 110% to 82% and stress falls from 0.17 N/m to 0.07 N/m.

The mechanical properties of kirigami WS2 nanosheets are significantly influenced by temperature, as demonstrated by the stress-strain curves across a range of temperatures from 100K to 600K. At lower temperatures, the kirigami WS2 structure retains higher stiffness and strength due to minimal atomic vibrations, resulting in a more stable lattice capable of withstanding greater stress before failure. As temperature increases, several factors contribute to a decrease in mechanical strength and earlier failure. Firstly, increased atomic vibrations lead to thermal expansion, which can introduce micro-cracks or defects that weaken the structure. Secondly, higher thermal energy weakens atomic bonds, making the material softer and more prone to deformation. Additionally, elevated temperatures enhance dislocation mobility, facilitating easier

deformation and reducing yield strength and ultimate tensile strength. The increased temperature also affects the flipping and rotation mechanisms in kirigami structures, as described in the referenced papers, by potentially altering the stress distribution and deformation pathways, leading to more localized failures. These combined effects result in the observed reduction in mechanical performance of kirigami WS2 nanosheets at higher temperatures.
